# Challenges with couples, serodiscordance and HIV disclosure: healthcare provider perspectives on delivering safer conception services for HIV-affected couples, South Africa

**DOI:** 10.7448/IAS.17.1.18832

**Published:** 2014-03-12

**Authors:** Tamaryn L Crankshaw, Deborah Mindry, Chantal Munthree, Thabo Letsoalo, Pranitha Maharaj

**Affiliations:** 1 Health Economics and HIV and AIDS Research Division (HEARD), University of KwaZulu-Natal, Durban, South Africa; 2 Department of Psychiatry and Behavioral Sciences, Center for Culture and Health, University of California, Los Angeles, CA, USA; 3 School of Built Environment and Development Studies, University of KwaZulu-Natal, Durban, South Africa

**Keywords:** safer conception, couples, serodiscordance, healthcare providers

## Abstract

**Introduction:**

Introduction Safer conception interventions should ideally involve both members of an HIV-affected couple. With serodiscordant couples, healthcare providers will need to manage periconception risk behaviour as well tailor safer conception strategies according to available resources and the HIV status of each partner. Prior to widespread implementation of safer conception services, it is crucial to better understand provider perspectives regarding provision of care since they will be pivotal to the successful delivery of safer conception. This paper reports on findings from a qualitative study exploring the viewpoints and experiences of doctors, nurses, and lay counsellors on safer conception care in a rural and in an urban setting in Durban, South Africa.

**Methods:**

We conducted six semistructured individual interviews per site (a total of 12 interviews) as well as a focus group discussion at each clinic site (a total of 13 additional participants). All interviews were coded in Atlas.ti using a grounded theory approach to develop codes and to identify core themes and subthemes in the data.

**Results:**

Managing the clinical and relationship complexities related to serodiscordant couples wishing to conceive was flagged as a concern by all categories of health providers. Providers added that, in the HIV clinical setting, they often found it difficult to balance their professional priorities, to maintain the health of their clients, and to ensure that partners were not exposed to unnecessary risk, while still supporting their clients’ desires to have a child. Many providers expressed concern over issues related to disclosure of HIV status between partners, particularly when managing couples where one partner was not aware of the other's status and expressed the desire for a child. Provider experiences were that female clients most often sought out care, and it was difficult to reach the male partner to include him in the consultation.

**Conclusions:**

Providers require support in dealing with HIV disclosure issues and in becoming more confident in dealing with couples and serodiscordance. Prior to implementing safer conception programmes, focused training is needed for healthcare professionals to address some of the ethical and relationship issues that are critical in the context of safer conception care.

## Introduction

The fertility desires of HIV-affected individuals are increasingly being acknowledged in the global public health setting, and a number of safer conception strategies exist that may reduce HIV transmission risk for serodiscordant couples who wish to conceive [1–5]. In South Africa, recent clinical guidelines recommend that services should support individuals living with HIV who wish to conceive and offer safer conception strategies for healthcare providers (HCPs) to draw on with recommendations to engage the client's partner [4]. The role of the provider is pivotal to delivery of safer conception services because they will need the necessary knowledge and technical skills to implement the service and, depending on their clinical perspectives and priorities, they will have direct influence on client access and uptake of care [5, 6]. Prior to widespread implementation of safer conception services and training of the relevant HCPs, it is crucial to better understand provider insights and experiences regarding provision of safer conception care [7, 8].

Safer conception interventions ideally involve both members of an HIV-affected couple. At the clinic interface, HCPs will need to tailor safer conception strategies according to the resources available and the HIV status of each partner within the relationship. Providers will also be required to provide advice to the couple for managing periconception HIV risk behaviour. Although the clinical strategies are arguably relatively easy to execute, dealing with the periconception HIV risk behaviour linked to these strategies is rather more complex and also needs to be understood and accounted for in safer conception interventions [9].

There is an additional consideration. In an effort to meet the goals of the current National Strategic Plan, South African nurses are being trained to conduct the bulk of antiretroviral (ARV) treatment initiations [10]. There has also been widespread utilization of lay counsellors and community healthcare workers to provide greater reach in linking patients to HIV-related care and to support HIV testing and ARV treatment compliance. Given the multidisciplinary approach to HIV care in South Africa, insight into the perspectives of a spectrum of healthcare provider categories is important because all levels of staff are likely to have significant client contact. Training requirements will differ among professional and layperson groups and will need to be tailored to address particular social norms, prior training, and medical culture [6].

This paper reports on some of the findings from a qualitative study exploring the perspectives of male and female doctors, nurses, and lay counsellors in a rural and an urban setting in Durban, South Africa, with specific attention to their experiences serving clients and their partners who have had or who have expressed a desire for children. This is a particularly relevant HIV prevention concern in KwaZulu-Natal, which has the highest antenatal HIV prevalence (37.4%) of all South African provinces [11].

## Methods

### Setting/study design

HCPs were recruited from two sites in Durban, South Africa. The first site was a public sector antiretroviral treatment (ART) clinic serving a large rural community. The clinic refers patients to other public sector health facilities for tuberculosis care, prevention of mother-to-child transmission (PMTCT) care, and for obstetric and gynaecological services. The second site was an ART clinic located at a state subsidized hospital serving a large urban and peri-urban population in the eThekwini district of the KwaZulu-Natal province. This site had developed in-house psychosocial questionnaires and clinic-based forms to support HCPs in providing comprehensive care. This site generally had greater resources available at the hospital including a PMTCT clinic, HIV paediatric care, and a male circumcision programme.

### Selection of participants

Fieldwork was conducted between May and October 2011. Providers were recruited through general invitation by researchers via staff meetings. We conducted six semi-structured individual interviews per site (a total of 12 interviews) as well as a focus group discussion (FGD) at each site (a total of two focus groups). The FGDs included a total of 13 participants and consisted of seven nurses and six lay counsellors working at the two sites. The individual interviews included five doctors, four nurses, and three lay counsellors. Individual interviews were conducted first to ensure that provider perspectives would not be influenced by the content in the FGD. Interviews were conducted in a private setting at each clinic site. Once individual interviews were complete, FGDs were conducted with nurses and counsellors; doctors were not included to avoid professionally based hierarchies that may have negatively affected participation by the counsellors and nurses.

Ethics approval was received from the University of California, Los Angeles; the University of KwaZulu-Natal, Durban; the state subsidized Hospital Research Ethics Committee; and the KwaZulu-Natal Department of Health. Permission was obtained from both sites to conduct the study.

### Interviews

Focus groups and individual interviews investigated provider views on current practices and experiences related to delivering reproductive health (RH) services to people living with HIV (PLHIV), to meeting the needs of men as well as women, and to the feasibility of providing appropriate RH services to PLHIV. Questions also explored provider perspectives on the resources for and challenges of providing safer conception services to PLHIV within the current healthcare delivery system and investigated the training needs of HCPs. Interviews and FGDs were scheduled to avoid disruption of clinic services and to fit with provider schedules. Interviews were conducted by a male and female researcher; the male was a Zulu language speaker, and both researchers were fluent in English. Most interviews were conducted in English with Zulu language interviews conducted as preferred or needed. Individual interviews were on average 35–45 minutes, and FGDs were 45–60 minutes; they were audio–recorded, translated, and transcribed by the interviewers.

### Analysis

All interviews were coded in Atlas.ti using a grounded theory approach to develop codes. Coding was led by the second author [DM]. The coding scheme identified major themes and subthemes as they emerged within the data. The coding scheme was reviewed by the research team and was revised based on feedback in collaboration with the first author [TC]. The major themes were shaped a priori by the broader questions explored in the interviews as described earlier. In this paper, we focus specifically on data coded to provider concerns regarding couples, serodiscordance, and male partner involvement. In our results, we have selected quotations that represent the varied perspectives of providers at the two different sites.

## Results

A subsection of the overall study findings is presented here. The specific themes related to couples-based dynamics in the provision of safer conception services include HCP's anxieties around dealing with the clinical implications of serodiscordance between couples, negotiating HIV disclosure between couples, and involving men in HIV testing and accessing clinical care.

### Dealing with serodiscordance

Managing the clinical and relationship complexities related to serodiscordant couples wishing to conceive was flagged as a concern by all categories of health providers, particularly among nurses and counsellors.

It's very difficult especially when the female partner is tested positive and she went home and disclosed to the male partner. You encourage the female partner to bring the male partner, but when the male partner comes to the clinic and tests negative that's a big issue. It can even lead to divorce. We have a patient who is in the process of being divorced because her partner tested negative and she tested positive, so we are dealing with discordant couples and it's a problem, and we really need help. We need training. [Urban site, female counsellor]

Nurses and counsellors admitted to referring serodiscordant couples to specialist services for advice rather than providing safer conception guidance themselves. They expressed discomfort in promoting the limited unprotected sex strategy for safer conception because this directly conflicted with the prevailing safer sex messaging in which they had been trained.

Counsellor1: … we can refer them [serodiscordant couples] to the gynaecologist. So they can take over [dealing with safer conception].

Nurse2 (agreeing).

Counsellor2: Because at the end of the day we cannot encourage, if one is positive and one is negative, you cannot encourage them not to use a condom.

Nurse1: We always say condom, condom, and condom.

(All agree).

Counsellor2: Because they will come and say you encourage them not to use protection. We can't do that.

Nurse1: Condom is on top of the list. [Urban site, FGD]

Although counsellors and nurses did not seem to have a problem encouraging condoms in principle, one doctor raised the point that talking about the specifics of sex for conception purposes may not be an area that all providers would be comfortable discussing. This may be an especially sensitive issue for providers who lack adequate training in and who are personally uncomfortable discussing sexual matters. Although general clinical guidelines had been published in June 2011, the lack of training and specific direction around safer conception strategies directly impacted provider comfort with the topic:

There are no clear [safer conception] guidelines on what we should advise people. So, clear guidelines for nurses, counsellors, and doctors, as well as advice for doctors would be helpful. And make sure that everyone knows. I don't know if the counsellors have been formally trained in sexual issues, we have had kind of in-house training. I think those are the main things. Also, realizing that not everyone is comfortable talking about sexuality. One would assume that everyone in an ARV clinic is happy to talk about sex but maybe not everyone is. Maybe we need to see who is not too comfortable with talking about it, and we make sure clients get channeled to the right person. [Rural site, male doctor]

Providers also revealed that, in the HIV clinical setting, they often found it difficult to find a balance between their professional priorities, maintaining the health of their clients, and ensuring that partners were not exposed to unnecessary risk, while still remaining mindful of and supporting their client (and partner's) desires to have a child:

Well, then we make sure that we change them onto the nevirapine as opposed to [efavirenz]. We inform them when to come back, to address whether their partner is positive or not. A lot of the women say they have partners who say they have tested but haven't shown any proof or they don't want to test. So there's sort of this balance between they want to have children but at the same time you want them to use condoms especially if their partner does or doesn't know their status; invariably they are at risk of getting infected and then you have cross infectivity and a chance of resistance, etc. It's very often a difficult situation. It's kind of like priorities have to be met before reproductive health services. [Rural site, female doctor]

Linked to the concerns surrounding the clinical management of serodiscordant couples were the underlying relationship complexities as a direct result of serodiscordant status. This was an issue that lay counsellors and nurses most often faced.

Counsellor1: For me it's like an interrogation sometimes, because there is a question in the form [baseline psychosocial questionnaire]: were you with him when you were with the current partner, and she will say no, and then I will ask when did you break up with your previous partner, she will say in 2003 and when did you meet with the current partner? They will say in 2002, so you were with …?

Nurse4: We are asked by our patients How? Why? Why? Why?

Counsellor1: We have been sleeping together for 3 years …. and how is she positive and I'm negative?

All nurses agree. [Urban site, FGD]

The possibility of concurrent sexual partners was a difficult topic for providers to navigate because clients, who were aware this went against HIV prevention messaging at the clinic, did not readily acknowledge this reality.

### HIV-related disclosure concerns

Many providers expressed concern over dealing with the issue of disclosure of HIV status between partners, particularly when managing couples where a partner was not aware of the other's HIV-positive status and where they were reportedly expressing the desire for a child.

Yes we do have that kind of problem where the husband wants a child but does not know that the wife is HIV-positive. [Rural site, female nurse]

Unknown HIV status of the partner made it very difficult for providers to support a request to have a child safely:

Well, I think the biggest one is only dealing with one half of the couple, that's probably the biggest challenge because very frequently the women will say the husband or the partner doesn't even know his status and doesn't want to know his status. So you're working in the dark and that's difficult. [Rural site, female doctor]

Fears over physical or emotional abuse and relationship breakups as a result of encouraging disclosure were also raised.

Counsellor1: We are trained to ask them open-ended questions but at the end of the day, you will get the answer that they want to give, that will benefit them. There was one lady … who came with a partner. She tested positive and the partner tested negative. They were newly married. The male partner then divorced her. Then she met this new guy and fell pregnant. She said she will never disclose her status, because the last time she disclosed her status and the partner divorced her.

Counsellor2: That's what happened to one of our patients as well. She came to our clinic and she tested positive whilst she was a few months pregnant and then went home and disclosed to the male partner. The male partner said he was negative meanwhile he was a [ART clinic] patient. [Urban site, FGD]

Providers reported that they often encountered patients who had not disclosed their HIV-positive status to their partner or had found it difficult to ascertain if disclosure between partners had actually occurred.

Nurse4: And sometimes I wonder when we ask patients, have you disclosed to your partner, I wonder if they are even telling us the truth, because they know the questions that we are going to ask. (All nurses agree.) [Urban site, FGD]

Several strategies for dealing with disclosure issues were offered by the HCPs.

What I normally do is ask as now that they are on ARVs, do they have a partner and does the partner know your status. If the patient is scared of telling the partner I ask them to come with their partner, then I pretend that I had seen her for the first time and when the results come out they will find out together. Because if I tell the partner or she tells her partner they end up fighting and the one who tested first is more likely to be blamed for bringing the virus. [Rural site, female counsellor]

Interestingly, one nurse viewed safer conception services as a potentially “safe” forum to address couples-specific issues.

Nurse3: If the preconception clinics are sort of being motivated, not just here but everywhere, it might just open a way where people will find it safe and also to start disclosing to their partners. So that it becomes like a joint effort, where “we” both decide about the family. [Urban site, FGD]

### Male partner involvement

Providers agreed that it was important to include both partners in safer conception care but highlighted that it was most often women who came in for care and that historically it had proven difficult to not only reach the male partner but also to include him in the consultation. The doctors in the urban and rural clinics related similar experiences between client genders:

Because it is a general trend, even in the hospital that the men wait until they are very sick before they come. The women also hold on and do not speak to their partners because they feel threatened in that relationship that he would leave her. [Urban site, female doctor]

… it's most often the case that the partner isn't here or living elsewhere, a woman doesn't know her partner's status. [Rural site, male doctor]

I would advise her … to bring her partner but then very often they promise to bring their partner, but you see them a few months later already pregnant without consulting us. Sometimes they think we are telling them not to fall pregnant. [Urban site, female doctor]

Providers suggested some potential barriers to men being included in care. Structural barriers included clinic access for men who were employed and financial constraints. Reported reluctance of the male partner to ascertain his HIV status or using his partner's status as proxy was a further barrier:

I think it would be nice for men to be involved because men don't usually come to the clinic, they assume that if the partner is HIV-positive then they are more likely to be positive so I don't know how men can be encouraged to come to the hospital. [Rural site, female counsellor]

Obviously for a lot of people it's such that you come at 7[o'clock] in the morning and you only get to see the Doctor at 1[o'clock] or 2 [o'clock] in the afternoon, and it's a whole day wasted [for the partner], and it's a financial burden. [Rural site, male doctor]

The fact that health providers were predominantly females was also raised as a gender barrier. Men may not feel comfortable discussing their concerns with a female health provider:

I think that there is not enough time and often men feel uncomfortable. All the counsellors in our clinic are women, except for one. All the nurses are women, and we have one male doctor other than me. So I'm sure that men do raise the issue with women, but I think it's more difficult. [Rural site, male doctor]

A counsellor recalled encountering this difficulty in the course of her work:

And when you are talking to a man, you can tell that some men don't want to listen to a female. If you are female health worker and you are trying to help, he will just be looking at you. [Urban site, FGD]

Some providers offered suggestions for creating an environment that may be supportive of male attendance.

I think you have to have a more healthy, friendly environment, you know what I mean. Almost like a couple services, like we have services for the kids. Maybe it could be encouraged to have couple testing, and for couples to come together with their partners. [Rural site, male doctor]

## Discussion

Research has shown that HCPs do not regularly engage in conversations around pregnancy plans and fertility desires, but few studies have actually sought out the perspective of HCPs themselves [6, 12–14]. The HCPs who participated in our study raised two primary concerns in terms of engaging couples in safer conception care. They expressed anxiety related to the clinical management of serodiscordant couples and concerns about dealing with the interpersonal relationship complexities surrounding HIV disclosure. Safer conception services will necessarily need to focus on the couple and will require HCPs to be familiar with the clinical management of HIV-affected couples as well as to be able to navigate some of the sensitive gendered and relationship elements related to couples [9, 15, 16]. As the study findings highlighted, the HCPs were most familiar with the traditional HIV prevention messages that counsel clients to avoid any risk through practicing safe sex. The suggestion of deliberate exposure to risk in an effort to conceive through unprotected sex (even if limited) will feel counterintuitive for many of these same providers, especially those categories of professionals who are trained to follow clinical algorithms, such as nurses, or those who do not have a formal clinical qualification, such as lay counsellors. This safer conception strategy is particularly relevant to resource constrained settings because limited unprotected sex, and not the expensive sperm washing alternative, will likely be a more realistic option.

Dealing with the unique constellation of relationship complexities among serodiscordant couples and negotiating HIV disclosure within those relationships was flagged as a particular area of concern by providers in our study. These providers described negative outcomes that clients had experienced as a result of disclosure, which led to some clients being reluctant to further disclose their status to another. Health providers may experience personal conflict and ethical difficulties in managing a serodiscordant couple where HIV disclosure has not occurred and will need specific guidance on how to deal with this possibility. In the case where one partner's HIV status is unknown, providers will need to address partner uptake of HIV testing as well as disclosure. Providers in our study expressed concerns regarding gender-based norms that affect clients’ willingness to disclose their status. A basic awareness of gender-based vulnerabilities, the risk of intimate partner violence, and of cultural expectations and norms related to fertility and pregnancy are also advised. Provider concerns around the potential consequences of HIV disclosure are valid and are supported by substantial research findings. Research has highlighted that breakdown or dissolution of a relationship [17, 18], economic abandonment, rejection, intimate partner violence, and isolation [18–22] are among some of the negative consequences experienced by women living with HIV following HIV disclosure to a male partner.

The difficulties in involving men in HIV testing and safer conception care was another challenge raised by providers in our study. Male partners may resist participating in couples counselling if the relationship is insecure or as a means to avoid relationship conflict [23–25]. Including men in the safer conception consultations presented a specific challenge to delivering safer conception services in our study, but the reality is that men are involved in reproductive decision-making and are intrinsically part of the safer conception process [5, 26–28]. Male partner involvement in antenatal and/or postnatal care or agreeing to undergo an HIV test has been associated with safer sex practices [29], better PMTCT outcomes [30, 31], increased adherence for female partners on ART, and improved communication between couples [29]. However, an understanding of gender power differences and how these may affect female decision-making regarding pregnancy is crucial [9]. Interestingly, a recent study cautions against a blanket approach to male involvement during and after pregnancy because it may not necessarily be in the best interests of the women, especially if there are difficulties around HIV disclosure [32].

The study findings suggest that because of their reported anxieties, some providers may avoid engaging with serodiscordant couples and may instead refer these clients to specialist or niche service providers. If safer conception services are to be scaled up and made accessible to all HIV-affected couples desiring a child, all levels of health providers need to be trained so that they are comfortable with providing safer conception care to serodiscordant couples. Although clinical guidelines for safer conception are available in South Africa [4], specific training in safer conception services with a view to implementation of the service is not yet routine. Prior to the widespread availability of safer conception services, it is important for public health specialists and programme implementers to consider the framing of messages for HCPs who will be delivering the service. Training in safer conception methods with a view to engaging couples and to challenges related to HIV serodiscordance is required. Safer conception services that are supported by well-trained and knowledgeable providers potentially offer safe spaces for couples to negotiate the impact of HIV serodiscordance on their relationships. In cases where couple dynamics do not allow for effective and safe implementation of the service, individual counselling strategies need to be considered.

## Limitations

There are some limitations to this study. Both study sites were selected based on the specific interest in provision of safer conception services, which meant that the participants were likely to have a greater understanding of the surrounding issues compared to other providers at different healthcare institutions. Also, given the professional hierarchies that exist among different categories of health providers, we cannot discount the influence of social desirability bias within the focus group interviews. Lastly, provider perspectives on safer conception care are only one part of the picture. Knowledge of patient perspectives is also required prior to the development of appropriate interventions.

## Conclusions and recommendations

It is recommended that prior to implementing safer conception programmes, training is provided to HCPs to address some of the ethical and relationship issues arising out of the specific context of care, including the strong possibility of intimate partner violence as a result of HIV serodiscordance between couples. Exploratory research within the relevant client population needs to be undertaken prior to training in order to identify the key dynamics arising out of the relationship and the social contexts that need to be addressed. Different categories of providers will require specific training on how to counsel couples and, in particular, how to effectively address disclosure issues in the context of safer conception.
